# Gonadal Steroids Negatively Modulate Oxidative Stress in CBA/Ca Female Mice Infected with *P. berghei* ANKA

**DOI:** 10.1155/2014/805495

**Published:** 2014-08-27

**Authors:** Néstor Aarón Mosqueda-Romo, Ana Laura Rodríguez-Morales, Fidel Orlando Buendía-González, Margarita Aguilar-Sánchez, Jorge Morales-Montor, Martha Legorreta-Herrera

**Affiliations:** ^1^Laboratory of Molecular Immunology, National Autonomous University of Mexico, FES Zaragoza Campus, 09230 México City, DF, Mexico; ^2^Department of Immunology, National Autonomous University of Mexico, Institute of Biomedical Research, University City, 04510 México City, DF, Mexico

## Abstract

We decreased the level of gonadal steroids in female and male mice by gonadectomy. We infected these mice with *P. berghei* ANKA and observed the subsequent impact on the oxidative stress response. Intact females developed lower levels of parasitaemia and lost weight faster than intact males. Gonadectomised female mice displayed increased levels of parasitaemia, increased body mass, and increased anaemia compared with their male counterparts. In addition, gonadectomised females exhibited lower specific catalase, superoxide dismutase, and glutathione peroxidase activities in their blood and spleen tissues compared with gonadectomised males. To further study the oxidative stress response in *P. berghei* ANKA-infected gonadectomised mice, nitric oxide levels were assessed in the blood and spleen, and MDA levels were assessed in the spleen. Intact, sham-operated, and gonadectomised female mice exhibited higher levels of nitric oxide in the blood and spleen compared with male mice. MDA levels were higher in all of the female groups. Finally, gonadectomy significantly increased the oxidative stress levels in females but not in males. These data suggest that differential oxidative stress is influenced by oestrogens that may contribute to sexual dimorphism in malaria.

## 1. Introduction

*Plasmodium* species cause more than 200 million cases of malaria each year, with more than 1 million deaths [1]. The mechanisms that are involved in immunity to malaria are extremely complex, with host and parasite factors influencing infection outcomes [2, 3]. Although the prevalence of* P. falciparum* infection does not differ between the sexes, parasite density is 2-fold higher in postpubescent (aged 8–46 years) boys than in girls, suggesting that circulating sex steroids may influence outcomes. Furthermore, sex differences in response to malaria infection have been reported among both adults and children [4–6]. In general, there is evidence that sex-associated hormones can modulate immunity and consequently influence the outcomes of parasitic infections. Males are generally more susceptible to infectious diseases compared with females [7–9]. Under normal circumstances, the levels of sex-associated hormones do not only differ between males and females but also vary according to age and pregnancy progress [10], and the severity of malaria infection is affected by these factors [7]. Specifically, testosterone and 17*β*-oestradiol are critically involved in the control of sexual dimorphism, affecting the immune response [11]. Typically, oestrogens depress T cell-dependent immune functions and aggravate B cell-dependent diseases, while androgens suppress both T and B cell immune responses. Furthermore, glucocorticoid stress responses, including immune challenges, are enhanced by oestrogens and strongly inhibited by androgens [12, 13].

The elimination of the* Plasmodium* parasite is associated with the increased synthesis of free radicals, which is induced by the immune response or by antimalarial drugs [14, 15]. Testosterone has been shown to increase the production of nitric oxide (NO) in macrophages [16] and has, therefore, been suggested to be a contributing factor to the sexual dimorphism of the immune response [12, 17].

Reactive oxygen species (ROS) are initiators of tissue damage and can upregulate enzyme activity [18]. Antioxidant defences work synergistically to maintain a redox balance. The detoxification of the superoxide anion and hydrogen peroxide, which are catalysed by intracellular superoxide dismutase (SOD), catalase, and glutathione peroxidase (GPx) enzyme activities, represents a major line of defence [19]. Oestrogens have been shown to upregulate* in vitro* antioxidant activities for membrane phospholipid peroxidation [20]. In malaria, redox status alterations contribute to disease manifestation, including sequestration, cerebral pathology, anaemia, respiratory distress, and placental malaria. In addition, the host immune response to malaria involves phagocytosis and the production of NO and oxygen radicals which also contribute to the pathology of the disease [21]. However, it is not yet clear whether sex steroids modulate the antioxidant enzyme system in malaria-infected individuals.

Gonadectomy is a widely used strategy to analyse the importance of sex steroids in influencing sex dimorphism [11]. In this work, we decreased the levels of gonadal steroids using gonadectomy to study their roles in oxidative stress mechanisms in CBA/Ca mice that were infected with* P. berghei* ANKA.

## 2. Materials and Methods

### 2.1. Mice and Parasites

CBA/Ca mice were kindly donated by Dr. William Jarra (National Institute for Medical Research, London, UK). The mice were bred, fed, and maintained in a specific pathogen-free environment at the FES Zaragoza Universidad Nacional Autónoma de México animal house facilities in accordance with the institutional and national official guideline NOM-062-ZOO-1999 for the use and care of laboratory animals.

*Plasmodium berghei* ANKA parasites were also kindly donated by Dr. William Jarra and were cryopreserved under liquid nitrogen. The parasites were thawed and immediately injected in one mouse; five days later, parasitised blood was obtained to infect CBA/Ca mice. All of the infected animals received an intravenous (i.v.) inoculation of 1 × 10^3^ *P. berghei* ANKA-infected erythrocytes.

### 2.2. Ovariectomy

One-month-old female mice were anaesthetised with ketamine (80 mg/kg [body mass])-xylazine (8 mg/kg [body mass]) (Phoenix Pharmaceutical Inc., St. Joseph, Missouri, US), and incisions were made in the lower abdomens. The ovaries were removed, and the abdomen was sutured. Sham-operated mice underwent an identical procedure without the removal of the ovaries. The mice were given 4 weeks to recover from the surgery and then infected with 1 × 10^3^ erythrocytes that were parasitised with* P. berghei* ANKA. The mice were sacrificed 9 days following infection, and the lack of ovaries was confirmed by visual inspection.

### 2.3. Orchiectomy

CBA/Ca male mice were castrated at 3 to 4 weeks of age. The mice were anaesthetised and the testes were pulled out through scrotal incisions. The ductuli efferentes were transected by electrocauterisation, and the testes and epididymis were removed. Sham-operated mice underwent an identical procedure without the removal of the testes.

Mice were allocated into three different groups: (1) intact mice, (2) mice that were bilaterally gonadectomised (Gx), and (3) mice that underwent surgery without gonadectomy (sham-operated). Gx and sham-operated animals were given 4 weeks to recover from surgery prior to parasite infection. The mice were sacrificed by cervical dislocation at 9 days after infection (p.i.), and blood and spleen samples were obtained to assess the antioxidant enzyme activities of SOD, GPx, and catalase as well as to assess NO and malondialdehyde (MDA) levels.

### 2.4. Parasitaemia

Three days after infection, thin blood smears were prepared daily, fixed with methanol, and stained with a 1 : 10 dilution of Giemsa stain (Sigma, St. Louis, MO, US) in water. Enumeration of the parasitaemia load was performed under a 100x oil immersion lens using a Zeiss Standard 20 microscope (Cark Zeiss LTD, Welwyn Garden City, UK). Parasitaemia levels of 0.5% and above were determined by counting the number of parasitised erythrocytes that were present in a total of 200 red blood cells. Lower levels of parasitaemia were assessed by counting the number of parasitised erythrocytes that were present in 50 fields. The course of infection in each group is shown as the geometric mean of the percentage of parasitaemia.

### 2.5. Haemoglobin Concentration (Hb)

Hb was measured by diluting 2 *μ*L of blood in 498 *μ*L of Drabkin solution (1 g NaHCO_3_, 0.1 g K_2_CO_3_, 0.05 g KCN, and 0.2 g K_3_Fe (CN)_6_ in 1 litre of distilled H_2_O). The amount of cyanomethaemoglobin formed was detected at 540 nm using a spectrophotometer and converted to mg/mL using a standard curve of rat Hb (Sigma). Measurements were taken on day 0 of infection and then daily (at the same time of day) from day 3 to day 9 after infection.

### 2.6. Specific Activity of Superoxide Dismutase (SOD)

SOD activity was evaluated using the RANSOD kit (Randox Laboratories, Antrim, UK). This kit uses xanthine and xanthine oxidase to generate superoxide radicals, which then react with 2-(4-iodophenyl)-3-(4-nitrophenol)-5-phenyltetrazolium chloride to form a red formazan dye. SOD activity was measured by the degree of inhibition of the reaction. On day 9 after infection, the mice were sacrificed, and 125 *μ*L of blood was collected in a heparinised tube. The samples were washed four times with 1 mL of 9.9% NaCl solution and then centrifuged. The pellet of erythrocytes was then brought up to 0.5 mL with cold redistilled water, mixed, and left to stand at 4°C for 15 min. The lysates were diluted 25-fold with 0.01 mmol/L phosphate buffer pH 7.0, and 12.5 *μ*L of the solution was reacted with the substrates and xanthine oxidase to measure SOD activity levels. All of the steps were performed according to the manufacturer's instructions (Randox Laboratories, Ltd., Crumlin, UK). The reaction kinetics was measured at 505 nm. The inhibition of the amount of chromogen that is produced is proportional to the SOD activity level in the sample. A 50% inhibition is defined as one unit of SOD, and the specific activity is represented as units per mg of protein.

### 2.7. Specific Activity of Glutathione Peroxidase (GPx)

To analyse the GPx activity, we used a method based on the oxidation of glutathione (GSH) by cumene hydroperoxide, catalysed by GPx, in the presence of glutathione reductase and NADPH. Oxidised glutathione (GSSG) is immediately converted to its reduced form with the concomitant oxidation of NADPH to NADP^+^. Two microliters of heparinised mouse whole blood was diluted with 498 *μ*L of Drabkin reagent to quantify the haemoglobin level, and 50 *μ*L of blood was treated according to the manufacturer's instructions (Randox Laboratories). The GPx activity levels were calculated using the decrease in absorbance at 340 nm using an UV spectrophotometer.

### 2.8. Specific Activity of Catalase

Catalase activity in the erythrocytes was assessed according to a previously described method [22]. Briefly, peripheral heparinised mouse blood was centrifuged, and the pellet was combined with 4 parts of distilled water. The lysate was diluted 1 : 500 in phosphate buffer (50 mM, pH 7.4), and 1 *μ*L of the resulting solution was mixed with 500 *μ*L of 30 mM H_2_O_2_. The optical density of the reaction was immediately recorded at 240 nm (A1) and again 60 seconds later (A2) using an UV spectrophotometer. A difference in absorbance/min at 240 nm was indicative of the presence of catalase activity. The results are shown as nmol of H_2_O_2 _consumed per min/mg protein (haemoglobin).

### 2.9. Nitric Oxide Quantification

Nitrate concentrations were evaluated according to the Griess method, as described previously [23]. Fifteen microliters of serum or homogenised spleen tissue from each mouse was incubated for 3 hours at room temperature with 5 *μ*L of nitrate reductase (5 U/mL; Boehringer Mannheim, Laval, Quebec, Canada) and 15 *μ*L of NADPH (1.25 mg/mL; Boehringer). Following incubation, 100 *μ*L of Griess reagent (1% sulphanilamide, 0.1% N-1-naphthylethylenediamine dihydrochloride, and 1% orthophosphoric acid; Sigma Chemical Co. St. Louis, MO, USA) and 100 *μ*L of trichloroacetic acid (10% aqueous solution) were added, and this mixture was incubated for 10 min at room temperature. Subsequently, protein precipitates were removed by centrifugation at 14,000 rpm for 5 min. Next, 100 *μ*L of each supernatant was transferred to a 96-well flat-bottom plate. Concentrations were evaluated in an ELISA reader (Stat Fax Plate Translator, USA, to 540 nm) using a standard curve with sodium nitrate (Sigma), which was diluted in similarly prepared pooled sera from uninfected control CBA/Ca mice.

### 2.10. Malondialdehyde (MDA) Analysis for Lipid Peroxidation

MDA levels were measured as previously described [14]. Briefly, homogenised spleen or blood samples equivalent to 1 mg of protein or standard (1,1,3,3-tetramethoxypropane (Sigma)) were combined with 100 *μ*L of orthophosphoric acid (0.2 M, Sigma), 125 *μ*L of BHT (2 mmol/L; Sigma), and 12.5 *μ*L of TBA (Fluka Chem, Buchs, Switzerland; 0.11 M in 0.1 mol/L NaOH (Sigma)). Both samples and standard were placed in a water bath, heated for 45 min at 90°C, ice-cooled to stop the reaction, and then extracted once with 250 *μ*L of n-butanol (Sigma). The butanolic phase was separated by centrifugation at 1,500 g for 3 min and the absorbance at 535 nm was measured in a spectrophotometer. The concentration of MDA was calculated using a calibration curve.

### 2.11. Statistical Analysis

Significant differences between the groups for activities of superoxide dismutase, glutathione peroxidase, and catalase were determined using a one-way analysis of variance. The results are presented as the means ± standard deviations. Statistically significant differences were considered when *P* < 0.05 using the Stat Graphics program for Windows, release 4.

## 3. Results

### 3.1. Gonadectomy Modifies Parasitaemia in CBA/Ca Mice Infected with* P. berghei* ANKA

**Figure 1:**
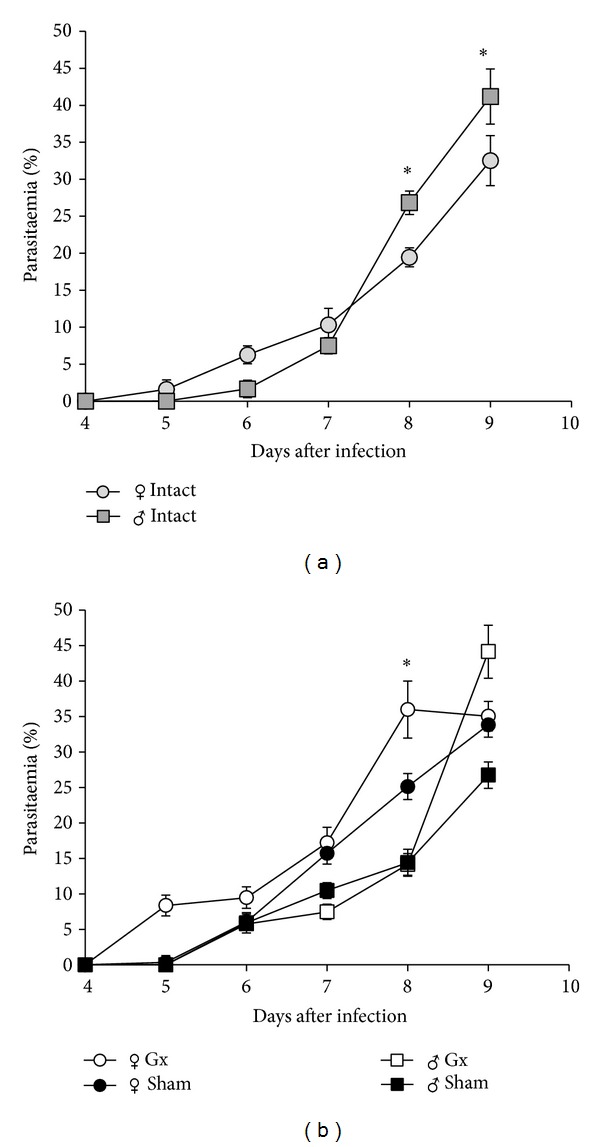
Effect of gonadectomy on parasitaemia levels in CBA/Ca mice infected with* P. berghei* ANKA. Gonadectomised, sham-operated, and intact female and male mice were infected with* P. berghei* ANKA. Parasitaemia levels were measured using Giemsa-stained blood films. Values are presented as the geometric mean ± SD (*n* = 10). Data are representative of two independent experiments. *indicates significant differences (*P* < 0.05).

To determine whether gonadal steroids influence differences in parasitaemia between female and male mice, groups of intact, sham-operated, and gonadectomised (Gx) male and female CBA/Ca mice were infected with* P. berghei* ANKA. Parasitaemia was monitored daily by Giemsa-stained blood smears. Intact female mice developed higher levels of parasitaemia than male mice between days 5 and 7 after infection. However, after day 7, the opposite effect was observed; male mice developed significantly higher levels of parasitaemia than female mice (Figure 1(a)). Gonadectomy significantly increased parasitaemia in female mice compared with sham-operated female mice. In contrast, gonadectomy in male mice only increased parasitaemia levels on day 9 after infection (Figure 1(b)).

### 3.2. Gonadectomy Influences Body Weight and Anaemia in CBA/Ca Mice Infected with* P. berghei* ANKA

**Figure 2:**
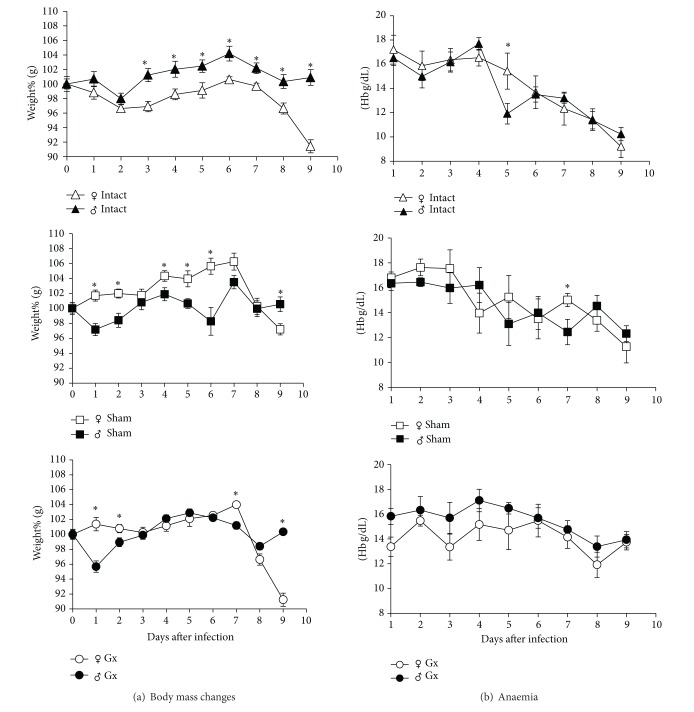
Effect of gonadectomy on body weight and anaemia in CBA/Ca mice infected with* P. berghei* ANKA. (a) Groups of gonadectomised, sham-operated, and intact female and male mice were infected with* P. berghei* ANKA; the weight on the day of infection (day 0) was considered to be 100%. Data represent the averages (means ± SD). *indicates significant differences (*P* < 0.05). (b) Haemoglobin levels were measured by the Drabkin method in the same groups of mice. Values are presented as the means ± SD (*n* = 10); *indicates significant differences (*P* < 0.05). Data are representative of two independent experiments.

Body mass and red blood cell turnover are affected by sex steroids [24–26]. In addition, cachexia is an important feature of malaria pathology [27]. Therefore, we evaluated whether the weight loss and anaemic differences that are typically observed between the sexes could be affected by reduction in the levels of gonadal hormones in* P. berghei* ANKA-infected mice. To this end, intact, sham-operated, and Gx female and male mice were infected and then weighed daily. The weight of the mice on the day of infection (day 0) was considered to be 100%, and weight loss or gain was expressed as a percent value compared with day 0. The intact male mice weighed significantly more compared with the intact female mice at the same age (8 weeks). The intact female group lost 4% of their body weight between days 0 and 2 after infection and then started to increase in weight until day 6, reaching 100%; however, after day 6, the mice displayed a continuous loss of body weight (Figure 2(a)). Interestingly, the sham-operated female group exhibited significantly higher weights than sham-operated male mice for the duration of the entire experiment. On days 8 and 9 after infection Gx female mice significantly decreased body weight compared with the sham-operated female group, corroborating the influence of gonadal hormones on the regulation of corporal mass. In contrast, Gx male mice showed no change in body weight compared with the sham-operated male group. Interestingly, sham-operated female mice had significantly increased body weights compared with both the infected intact and infected sham-operated female groups. These findings suggest that sexual hormones in females are associated with weight increase, while in males sex hormones do not modify body weight in mice infected with* P. berghei* ANKA (Figure 2(a)).

To address whether the anaemia that develops in mice infected with* P. berghei* ANKA differs between the sexes and whether it is influenced by the presence or absence of gonadal steroids, groups of intact, sham-operated, and Gx female and male mice were infected with* P. berghei* ANKA. The peripheral blood concentrations of haemoglobin (Hb) in the mice were quantified daily. Parasitic infection decreased the levels of Hb in all groups. Both intact female and male mice exhibited lower Hb concentrations on days 8 and 9 after infection, and no significant differences were detected between the groups. In general, gonadectomy inhibited the decrease of Hb in the infected mice, but the Hb levels were significantly higher in Gx male mice compared with Gx female mice (Figure 2(b)).

### 3.3. Gonadectomy Decreases the Specific Activities of Catalase, SOD, and GPx in Both Female and Male Mice

The malaria parasite is highly susceptible to oxidative stress [21]. The mechanisms of the host defence system include phagocytosis and the production of NO and oxygen radicals, which modify the oxidative stress levels during infection. Certain enzymes work as free radical scavengers, which can circumvent the oxidative stress that malaria parasite induces in the host. These enzymes include catalase, SOD, and GPx, which are considered to be some of the most important host protective enzymes [28]. Therefore, to determine whether gonadal steroids could differentially affect oxidative stress levels caused by the parasite, the specific activities of catalase, SOD, and GPx were measured in the blood and spleen of intact, sham-operated, and Gx male and female CBA/Ca mice that were infected with* P. berghei* ANKA.

**Figure 3:**
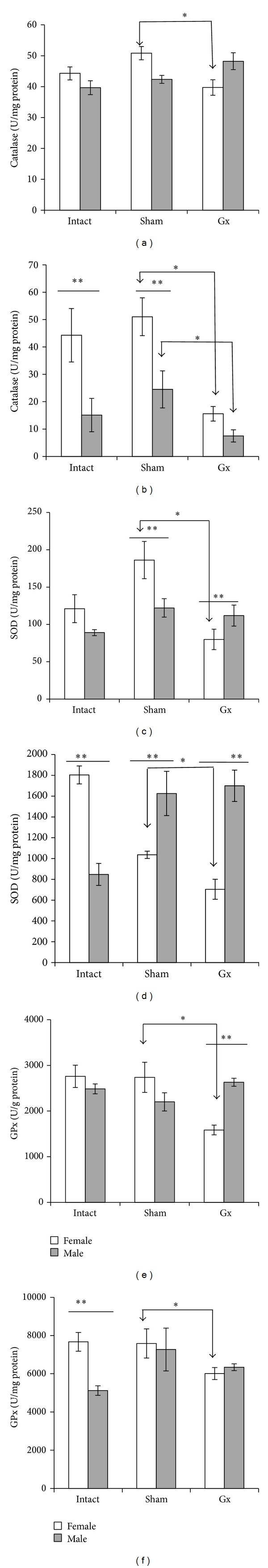
Gonadectomy decreases the specific activity of SOD, GPx, and catalase in female mice infected with* P. berghei* ANKA. Gonadectomised, sham-operated, and intact female and male mice were infected with* P. berghei* ANKA, and on day 9 after infection, the mice were sacrificed, and blood (a) and spleen (b) samples were obtained to quantify the specific activities of catalase, SOD, and GPx. Data represent the average (means ± SD). (*n* = 10), *indicates significant differences (*P* < 0.05). **indicates significant differences between female and male mice. Data are representative of two independent experiments.

Intact female mice exhibited higher specific activities of catalase than intact male mice; however, this difference was only significant in the spleen (Figure 3(b)). Gonadectomy decreased the specific activity of catalase in both the blood and the spleen of female mice compared with female sham-operated mice. Gonadectomy in male mice did not significantly modify catalase activity in the blood (Figure 3(a)). In contrast, catalase activity was significantly decreased in the spleen of Gx male compared with sham-operated male mice (Figure 3(b)).

Intact female mice had higher SOD activities than male mice in both the blood and the spleen tissues. These activities were reduced in the blood and spleen of Gx female mice relative to sham-operated female mice. In contrast, no significant alterations in specific activity were detected in Gx male mice in the blood or spleen compared with sham-operated male mice (Figures 3(c) and 3(d)).

Intact female mice displayed slightly higher GPx activities than intact male mice. Gonadectomy reduced these activities in female mice but did not modify them in the blood of male mice compared with the corresponding sham-operated group (Figure 3(e)). Gonadectomy significantly reduced the specific activity of GPx in the spleen of female mice but showed no effect in the spleen of male mice (Figure 3(f)).

### 3.4. Gonadectomy Increases the Levels of NO in Female Mice

**Figure 4:**
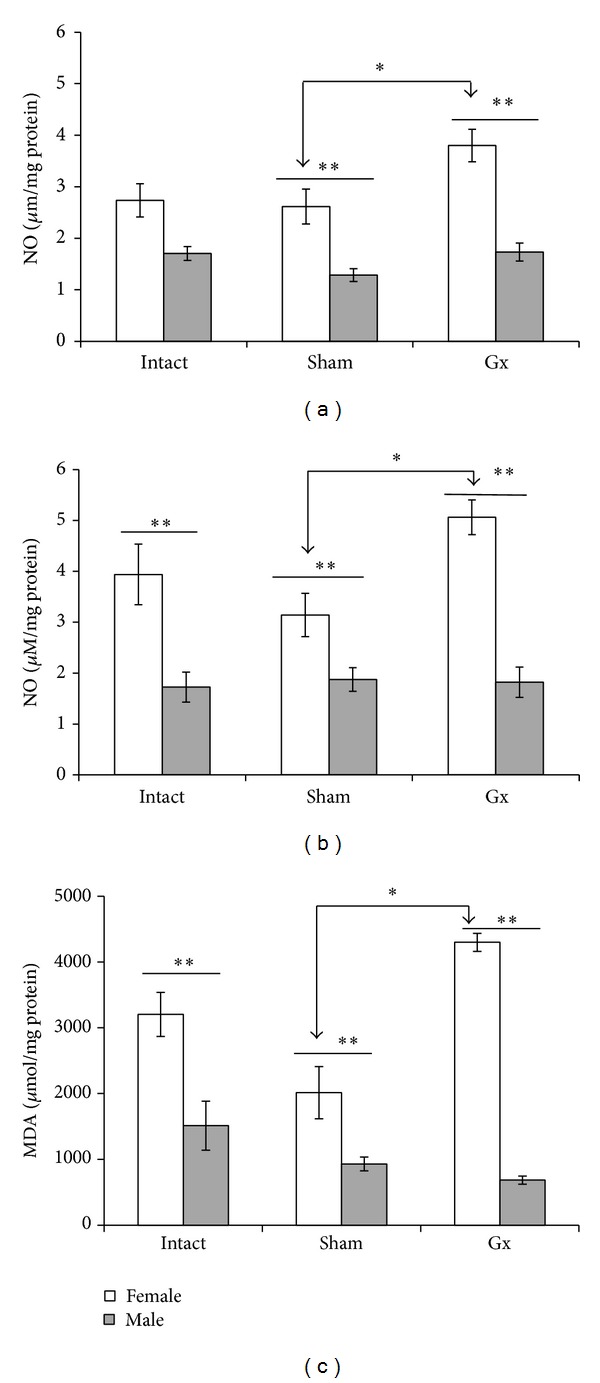
Effect of gonadectomy on nitric oxide and MDA in* P. berghei* ANKA-infected mice. Gonadectomised, sham-operated, and intact female and male mice were infected with* P. berghei* ANKA, and on day 9 after infection, mice were sacrificed, and blood (a) and spleen samples (b and c) were used to quantify the levels of NO and spleen was used to measure the concentration of MDA. Data represent the average (means ± SD). (*n* = 10), *indicates significant differences (*P* < 0.05). **indicates significant differences between female and male mice.

NO in the presence of oxygen is oxidised to different biologically active nitrogen oxides known as reactive nitrogen intermediates (RNIs). NO is a potent immune-modulator that has been implicated in both immune response against the malaria parasite [15] and malaria pathology [29]. Therefore, we analysed whether gonadal steroids could differentially affect the NO levels between sexes in CBA/Ca mice infected with* Plasmodium*. To this end, intact, Gx, and sham-operated female and male mice were infected with* P. berghei* ANKA, and on day 9 after infection, NO levels were measured in both serum and spleen tissue cells using the Griess reaction. All of the groups of female mice showed significantly higher levels of NO compared with the respective male groups. Gonadectomy significantly increased NO levels in the serum and spleen of female mice compared with sham-operated female group. Gonadectomy in male mice had no effect on NO levels in the blood or spleen (Figures 4(a) and 4(b)).

### 3.5. MDA Levels

MDA levels were measured as a proxy for the extent of lipid peroxidation in the spleen, the organ in which the malaria parasite is ultimately eliminated. MDA concentrations were significantly higher in intact female mice compared with intact male mice. Gonadectomy in female mice significantly increased the MDA levels in the spleen compared with the sham-operated female group. In contrast, gonadectomy in male mice decreased the MDA levels; however, this difference was not statistically significant compared with the sham-operated male group (Figure 4(c)).

## 4. Discussion

The results of the present study show for the first time that gonadal steroids modulate oxidative stress in* P. berghei* ANKA-infected mice. Gx female mice that were infected with the parasite developed significantly higher oxidative stress levels compared with their male counterparts.

The hormonal effects that occur as a result of gender differences do not only affect growth but also exhibit effects on host immunity. After* Plasmodium* infection, the immune response increases phagocytosis and the production of oxygen radicals and NO, which play key roles in host defence mechanisms against malaria [21] because* Plasmodium* parasites are highly susceptible to oxidative stress [30]. However, the relationship between the redox status of malarial parasites and that of their host is complex, involving both the antioxidant and host defence systems. In fact, the generation of oxidative stress is an important chemotherapeutic strategy against the malaria parasite [14, 31, 32]. However, redox status alterations also contribute to disease manifestations, including sequestration, anaemia, and cerebral pathology [33]. Nevertheless, the relationship between the immune system's oxidative stress response to malarial parasites and gonadal hormones that are involved in sexual dimorphism is complex and has not been elucidated in the parasitised host.

The present study shows that the infection of CBA/Ca mice with* P. berghei* ANKA induced higher parasitaemia levels in intact males compared with intact female mice. However, both anaemia and parasitaemia were exacerbated in Gx female compared with Gx male mice. These findings suggest an involvement of gonadal steroids in the control of parasite proliferation and erythropoiesis in female CBA/Ca mice. In general, anaemia is characterised by the reduction in Hb levels in relation to age, gender, and physiological status. However, the aetiology of anaemia in malaria-infected individuals includes the lysis of infected and uninfected red blood cells [34], dyserythropoiesis, and bone marrow suppression [35, 36]. The increased anaemia that was observed in infected Gx female mice could be potentially explained by their increased NO levels because it has been shown that NO inhibits haem synthesis and iron uptake via the transferrin receptor pathway [37]. In addition, the cytokines TNF-α and IFN-*γ* induce the suppression of human haematopoiesis, which is mediated by NO [38]. Finally, anaemia could be also increased by the lysis of parasitised erythrocytes.

Sex hormones affect oxidative stress levels. For example, changes in the physiological levels of oestradiol and testosterone alter the production of O_2_^−^ and H_2_O_2_ in rat macrophages [39]. In addition, the levels of 8-oxo-7,9-dihydro-2′-deoxyguanosine and other modified bases are higher in males than in females [40]. In the present study, the effect of gonadal hormones on oxidative stress in* P. berghei* ANKA-infected mice was supported, particularly in the Gx female mice group, by the significantly decreased catalase, GPx, and SOD specific activities in both blood and spleen of infected female mice compared with sham-operated female or Gx male mice. These findings could be explained by the fact that oestradiol increases the antioxidant activities of these enzymes [41].

The increased NO concentrations that were detected in the blood and spleen of the infected Gx female group correlated with the enhanced oxidative stress that was observed in these mice, particularly when compared with the sham-operated female or Gx male groups. The increased production of NO in* P. berghei* ANKA-infected Gx female mice may be explained by the fact that oestradiol inhibits nitrite release by macrophages [39]. However, even the intact group of infected female mice developed higher levels of NO compared with their intact male counterparts. This increase in NO levels in infected females is consistent with previous reports of female resistance to infection in experimentally induced malaria [42] and also supports the sexual dimorphism that has been described in malaria models [43]. However, our findings with regard to NO in the Gx female mice do not correlate with previous studies [44], and we believe that this divergence may be explained by variations in the numbers of parasitised erythrocytes in the inocula and in differences in the time points at which the NO levels were measured because we have found that NO peak levels only last for two days during infection [15].

In this work, increased lipid peroxidation levels as indicated by MDA concentration were observed in* P. berghei* ANKA-infected Gx female mice compared with both sham-operated female and Gx male groups. The decreased antioxidant enzymatic activity and increased levels of MDA could be explained by the fact that oestradiol, due to its hydroxyphenolic structure, may donate hydrogen atoms to lipid peroxyradicals to terminate chain reactions and act as an antioxidant and free radical scavenger [45]. In addition, oestradiol may upregulate the expression of the antioxidant enzymes catalase, SOD, and GPx [46].

Finally, testosterone suppresses immunity against* Plasmodium chabaudi* in C57BL/10 mice [47–49]. Consistently, testosterone administration induces a lethal outcome of otherwise self-healing blood-stage infections in female mice [50]. Supporting this finding, Gx male mice have been shown to be more resistant to* Plasmodium* infection [49]. These findings are consistent with the results of our previous study in which the administration of testosterone decreased the oxidative stress in female mice infected with* P. berghei* ANKA. This suppression could be partially explained by the fact that testosterone decreases the number of blood leukocytes [51]. The sexual dimorphism in malaria is complex, with oxidative stress being modulated by the direct antioxidant action of oestradiol or via regulation of gene expression. These processes can only partially explain the observed phenomena. Additional biological functions of gonadal hormones also play important roles, including the testosterone-mediated induction of immunosuppression.

## 5. Conclusions

Gender differences may significantly affect immunological responses. In this work, we presented evidence that* P. berghei* ANKA-induced oxidative stress is higher in female CBA/Ca compared with male mice and that gonadal steroids negatively modulate oxidative stress levels, mainly in female mice. These findings may partially explain the gender differences that are observed in terms of susceptibility to malaria and should be taken into account when considering treatment options or designing future effective vaccines against* Plasmodium*.
